# Digital-Based Nutrition Interventions Employing the Dietary Approaches to Stop Hypertension (DASH) Diet: A Systematic Scoping Review

**DOI:** 10.1155/ijhy/6175223

**Published:** 2025-08-30

**Authors:** Elizabeth Dodge, Patricia J. Kelly, Basil H. Aboul-Enein

**Affiliations:** ^1^Applied Nutrition Graduate Program, College of Professional Studies, University of New England, 716 Stevens Ave, Portland 04103, Maine, USA; ^2^College of Nursing, Thomas Jefferson University, 901 Walnut Street, Philadelphia, Pennsylvania, USA; ^3^Health & Society Program, College of Arts & Sciences, University of Massachusetts Dartmouth, 285 Old Westport Rd, Dartmouth 02747, Massachusetts, USA; ^4^Faculty of Public Health and Policy, London School of Hygiene & Tropical Medicine, 15-17 Tavistock Place, London WC1H 9SH, UK

**Keywords:** Dietary Approaches to Stop Hypertension (DASH), interventions, mHealth, nutrition, technology

## Abstract

**Background:** The Dietary Approaches to Stop Hypertension (DASH) diet is an internationally recognized anti-hypertensive dietary model. This systematic scoping review examines the effectiveness of digital-based interventions utilizing the DASH dietary pattern.

**Methods:** A search was conducted using 14 databases to include relevant studies from 1997 to January 2025 using PRISMA guidelines for scoping reviews.

**Results:** The review included 24 studies with almost 7000 participants, including randomized controlled trials and cohort studies conducted in several countries. Interventions using the DASH dietary pattern positively affected blood pressure (BP), nutrition behavior, and weight. Some studies also reported secondary outcomes such as reduced healthcare cost savings.

**Conclusion:** Technology-based DASH diet interventions yielded favorable health outcomes, particularly in reducing BP and dietary salt intake, as well as improved diet quality. This systematic scoping review supports the potential of digital-based interventions utilizing the DASH dietary pattern to improve nutrition and health outcomes, particularly those related to hypertension management. The findings emphasize the importance of using evidence-based approaches, which are grounded in theoretical frameworks and models to develop effective interventions, and thoughtful program design to maximize group effectiveness. Other factors that influenced the effectiveness of the intervention included the type of technology used, as well as participant comfort with using technology. Further research and development are needed to optimize these interventions for widespread impact and long-term sustainability.

## 1. Introduction

Hypertension affects one-third of adult populations and is regarded as the leading preventable cause of premature death worldwide [1–3]. While blood pressure (BP) is considered elevated at a measure of systolic blood pressure (SBP) of 120–129 mmHg and diastolic BP (DBP) of > 80 mm/Hg, stage 1 hypertension is diagnosed with an SBP of 130–139 mmHg and DBP of 80–89 mHG [4, 5]. The number of individuals living with hypertension doubled between 1990 and 2019, from 650 million to 1.3 billion [6, 7]. Worldwide, nearly half of the population presumed to have hypertension is unaware of their condition because high BP typically has no symptoms [8, 9]. Therefore, regular medical examinations are essential for prevention, diagnosis, and treatment. Hypertension is often referred to as a ‘complex disease' with genetic factors, lifestyle choices, and social determinants of health impacting incidence and management [10]. Ethnicity, education level, poor healthcare access, socioeconomic status, and living in high-poverty regions can all contribute to an increased risk of hypertension [10–12], as well as risk factors such as physical inactivity and unhealthful dietary patterns, particularly those that include large quantities of ultra-processed foods [1, 2, 13–17].

The Dietary Approaches to Stop Hypertension (DASH) dietary pattern was developed to prevent and manage hypertension and focuses on the consumption of vegetables, fruits, legumes, nuts/seeds, and whole grains while limiting salt, alcohol, and saturated fats [18–20]. Compared to a typical Western Diet, DASH diets tend to be higher in micronutrients, fiber, and plant-based protein and have a marked reduction in dietary salt intake (1500 mg/day) compared to the US average daily consumption of 3300 mg [21, 22], which can have positive effects on BP and overall health outcomes [20, 23].

Recent research has investigated the utility of digital-based and online platforms for the delivery of nutrition education and behavior change interventions [24–28]. Referred to as mHealth (mobile health) or eHealth (electronic health) delivery modalities, interventions that are offered digitally have demonstrated promise in improving health, nutrition, and lifestyle behaviors [29, 30]. However, several reviews have found that while many eHealth and mHealth interventions were considered accessible and low cost to implement, many were not rooted in evidenced-based nutrition guidelines [31–37]. Other reviews and studies have also found that eHealth and mHealth interventions are cost-effective, appealing to a broad section of the population, and more easily accessible than place-based interventions, particularly for those with lower incomes and across a variety of ethnicities [38–44]. The aim of this systematic scoping review is to evaluate the current literature on DASH digital interventions and the impact on nutrition and health behavior outcomes.

## 2. Methods

### 2.1. Search Procedures

**Figure 1:**
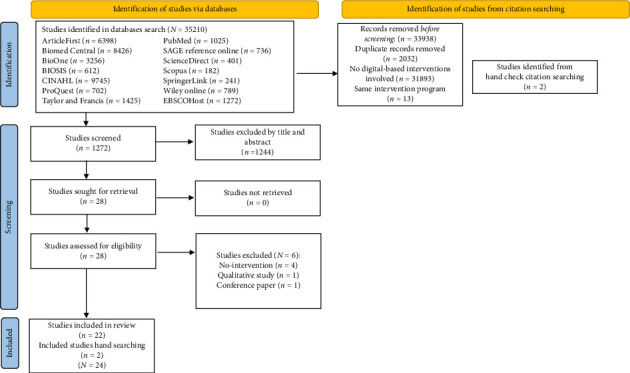
PRISMA 2020 flow diagram.

The search (see Figure 1) was conducted to include relevant studies from 1997, when the clinical data from the first DASH diet trial was published [45], to January 2025 and used PRISMA guidelines [46] and the framework of Arksey and O'Malley [47]. A comprehensive search was conducted using 14 academic databases—ArticleFirst; BioMed Central; BioOne; BIOSIS; CINAHL; ProQuest; Taylor and Francis; PubMed; SAGE Reference Online; ScienceDirect; Scopus; EBSCOHost; SpringerLink; and Wiley Online. Relevant studies were identified using a combination of the following terms: “online; app; digital; computer; technology; internet; web-based; intervention; program; education; DASH diet; DASH dietary pattern; DASH diet; hypertension; BP.” The search strategy was adapted according to the indexing systems of each respective database.

(See Supporting Information (available here)—Keyword search strategy applied to Scopus Database).

### 2.2. Selection Criteria and Study Quality Assessment

Article titles were screened for relevancy, and journal abstracts were reviewed. Studies were evaluated for relevance, merit, and inclusion/exclusion criteria (see Table 1). Reference lists of included studies were screened for additional relevant studies. Articles accepted for inclusion were individually reviewed by each author, and relevant data were extracted and tabulated (see Table 2). In order to investigate the effectiveness of digital-based nutrition and health behavior change interventions including the DASH dietary pattern, studies were eligible if they (1) included a digital-based intervention component such as an app, computer, internet web, smartphone, or any other technology-based approach, modality, or platform; (2) the digital intervention assessed any kind of nutrition behavior or outcome; and (3) the measured outcomes included nutrition or health-related outcomes, including nutrition behavior and physical activity. Feasibility studies with no outcomes were excluded. Methodological quality and bias assessment were conducted based on the 2022 Academy of Nutrition and Dietetics Evidence Analysis Manual Quality Criteria Checklist for primary research, and the rankings are included in Table 2 [48].

## 3. Results

In total, 24 studies including almost 7000 participants were evaluated for the systematic scoping review. Eighty-three percent (*n* = 13) of the studies included used a randomized study design [49–65], while the remaining designs included quasi-experimental and longitudinal/observational (*n* = 4) [66–69]. There was one small pilot study [70]. The majority of the studies were conducted in the United States (*n* = 16); two studies were conducted in New Zealand [57, 63], and one study was conducted in each of the following countries: Bangladesh [61], China [52], Iran [64], Japan [66], Saudi Arabia [70], and Thailand [71]. All studies included in this review utilized adult participant populations. In terms of participant health status, 50% (*n* = 12) of the studies were carried out with individuals with a diagnosis of prehypertension or hypertension [51–53, 56, 61, 64, 65, 68–72]. Three studies included patients with cardiovascular disease (CVD) or CVD risk factors [54, 55, 63], four studies included participants from the general community [58, 60, 66, 67], one study examined intervention effects in pregnant women [49], and one in women with a history of preeclampsia within the last 5 years [59], while another investigated mothers with a BMI > 24.9 [62]. Finally, one study examined adults with a BMI > 25 [50], and one enrolled participants with gout [57]. For the delivery of the interventions, thirteen of the studies utilized an app as the primary educational tool [53, 54, 57, 58, 60, 63, 64, 66, 68–72], seven were delivered via the internet [51, 52, 55, 56, 59, 62, 67], and four interventions were delivered via cell phone and/or mixed mobile health methods using health coaching prompts by location, SMS messaging prompts, or phone call follow-up [49, 50, 61, 65].

### 3.1. Digital DASH Interventions and Evidence-Based Approaches

Of the 10 studies that utilized a theoretical framework, model, or construct of behavior change [50, 56, 58, 60, 62, 68, 69, 73, 74], three used the social cognitive theory [50, 56, 73], one study used a combination of social cognitive theory and the social ecological model [74], one study each used motivational interviewing [69], self-determination theory [58], problem-solving skills [60], coaching strategies based on the human behavior-change approach [68], episodic future thinking [62], and the integrated model of health literacy [71]. Ten of the studies included some form of human interaction in the form of coaching [50, 51, 55, 58, 59, 68, 69, 74–76]. Study participants were followed for lengths of time ranging from one to two weeks [62] to 2 years [50]. The studies with the two longest follow-up periods of one and 2 years had strong research designs [50, 77].

Based on the quality assessment of the included articles, thirteen (13) were considered of positive quality [49–56, 59, 61, 65, 71, 72], eight (8) were neutral [57, 58, 60, 63, 64, 66–68], and three (3) had negative quality ratings [62, 69, 70]. The studies included with negative ratings were statistically underpowered pilot studies [62, 69], with one of very short (2-week) duration but with compelling intervention elements aligned with the inclusion criteria for this review.

Outcomes of interest varied across studies, with 15 of the 24 studies specifically noting BP as a study measure [50, 52, 53, 55, 59–61, 63–65, 67–69, 71, 72]. Other outcomes related to DASH dietary pattern adherence such as DASH score, Healthy Eating Index score, and DASH dietary components such as fruit and vegetable intake, potassium, magnesium, vitamin C were assessed across the studies. Physical activity was a frequently assessed outcome, with nine studies [50, 52, 56, 58, 59, 61, 64, 66, 69] reporting on steps per day, level of activity, or minutes spent in physical activity. Four of the studies [57, 63, 66, 69] also evaluated the level of engagement with the app or education modules.

### 3.2. Digital DASH Interventions and BP

The DASH dietary pattern was initially developed as a dietary approach to controlling hypertension, so it is logical that of the 24 articles reviewed, 15 digitally delivered DASH interventions included BP as an outcome [51–53, 55, 59, 60, 63–65, 67–69, 71, 72]. However, of those 15 interventions, only 10 recorded changes in BP [51–53, 59, 61, 64, 65, 67, 68, 71], and of those, only 5 studies [51, 64, 67, 68] found significant change postintervention, while the remaining four [53, 59, 61, 65] found either nonsignificant decreases in the intervention group or comparable decreases in both the intervention and control groups.

### 3.3. Digital DASH Interventions and Dietary Outcomes

Of the studies that reported dietary intake changes, three reported significant improvements. Moore et al. [67] observed that interventions led to a significant increase in the intake of fruits, vegetables, and grains (*p* < 0.05). The study by Schiwal et al. [58] found that diet quality improved significantly in individuals aged 58–64 years old (*p* < 0.03) after the intervention. Youngiam and M. Therawiwat [71] found a significant decrease in salt intake in the intervention group, while Jahan et al. [61] reported that both the intervention and the control group experienced a significant decrease in salt intake (*p* < 0.001).

## 4. Digital DASH Interventions and Physical Activity

The effect of the intervention on weight and BMI was reported in several studies. Moore et al. [67] found that after 12 months, the intervention resulted in a significant reduction in body weight by 4.2 lbs. (*p* < 0.001). Jerome et al. [76] reported a noteworthy decrease in body weight by approximately 10.6 lbs. (*p* < 0.05) following the intervention. Svetkey et al. [50] observed that the test group had significantly higher weight loss compared to the control group at 6 months (*p*=0.003), but this difference was not sustained at 12 and 24 months. Conversely, Van Horn et al. [49] noted a significant difference in weight gain in the intervention group, with an increase of 3.7 lbs. (*p*=0.01). Furthermore, Toro-Ramos et al. [68] demonstrated that among participants who completed the program with 80% commitment, there was a significant reduction in weight by 6.7 lbs. (*p*=0.001).

Some of the studies reported results showing changes in some related aspect of health. For example, the randomized controlled 9-month clinical trial of Rich-Edwards et al. [59] with women with a history of preeclampsia improved CVD risk knowledge and self-efficacy to achieve a healthy diet and reduced physical inactivity among this sample of women. Sacks et al. [77] conducted a study that showed a reduction in healthcare costs by $827 during the year-long intervention (*p*=0.05).

## 5. Discussion

Nutrition and health behaviors and dietary patterns are difficult to change [78–81]. Many theories, frameworks, and models of behavior change have been developed to encourage positive change in health behaviors, while discouraging behaviors that may negatively impact health, such as smoking, consuming too much sodium, or consuming a low nutrient density diet [80–85]. In concordance with other literature on health promotion interventions, interventions developed using evidence-based approaches to health behavior change show stronger outcomes. Such approaches ensure that the appropriate behaviors are targeted using educational and behavior change techniques that support the participant and allow the efficacy of the intervention to be assessed [85–90].

Dietary approaches to HPN control are important contributors to addressing the disease process, as are evidence-based approaches to behavior change. However, HPT is a long-term, complex disease with a variety of contributing mechanism, including diet in general and salt intake specifically, physical exercise, genetics, baseline weight and body composition, and overall health, including concurrent disease such as diabetes, COPD, renal failure, and/or psychiatric disorders. Interventions that focus on only one or two selected inputs to the HPT disease process for short intervals are less likely to see success in the critical distal outcomes of BP measurement and weight, even with the best of theoretical approaches.

The most important outcomes in studies focused on the contribution of DASH diets and HPT are actual changes in BP, which were recorded as an outcome in only 15 of the 24 identified studies [51–53, 55, 59, 60, 63–65, 67–69, 71, 72]. These changes are going to be difficult to achieve in studies with follow-up that is limited to a few weeks to months [51, 52, 54–56, 61–63, 65, 68]. Proximal outcomes such as knowledge and DASH scores are important for the feasibility of implementing complex and expensive research designs. However, the distal outcome of BP control and cardiac health must somehow be integrated into measuring the complex interactions of diet, disease, and longevity.

In addition to the body of health promotion literature that supports evidence-based approaches to intervention development, implementation, and evaluation, there is also considerable literature considering the effect of intervention delivery mode. Interventions that can be partially or wholly delivered through an app and/or are accessible on a phone or computer may be more accessible to participants, lower cost to researchers, and increase participation by fitting more easily into the busy lives of participants. However, intervention outcomes delivered through these modalities have been mixed [27, 91–94]. The interventions included in this review reflect the range of effect that digitally delivered DASH intervention can have on BP, DASH score, HEI score, and DASH dietary components. Of the 12 positively scored interventions, 11 found significant intervention effects in reduction of salt intake, intake of DASH diet components, increased knowledge on CVD risk factors and self-efficacy for healthy eating, DASH score, overall BP, SBP, weight reduction and gestational weight gain. One of the 11 studies found that the significant weight loss between the intervention and control group did not persist at 12 month follow-up [50]. Although not all included interventions had statistically significant findings, the overall trend toward positive health improvements across the studies indicates the potential for health and nutrition behavior change interventions that are evidence-based and technology-delivered. This is in line with other findings on digitally delivered dietary interventions, but the low-to-moderate quality of the overall evidence across the literature suggests a need for more research in this area [28, 95–100].

Interventions that utilized a personalized approach and/or that included virtual interaction with a member of the research team were in general more effective at achieving at least some of the stated outcomes, suggesting that participants may either find a mixed approach to intervention delivery more engaging or that they were more likely to feel accountable for participation. This is in alignment with the current literature on digital health promotion interventions [28, 95, 98, 101–104].

Based on the existing literature, well-planned and evidence-based approaches to digital health promotion interventions may be able to target and positively impact nutrition and health behavior. Such interventions appear to be most effective when they employ a behavior change framework or model, are culturally appropriate, and include some personalized and/or human interaction. The current review finds that there is a low to moderate impact of digitally delivered DASH interventions on a variety of health outcomes. Due to the heterogeneity of the studies and outcomes of interest, more research is warranted to determine the best approaches to engaging participants with digitally delivered DASH-focused interventions and for effecting change in intended outcomes.

## 6. Conclusion

This systematic scoping review supports the potential of digital-based interventions using the DASH dietary pattern to improve nutrition and health outcomes, particularly in increasing adherence to the DASH dietary pattern as measured by DASH score. More research is needed to determine how to best effect change in BP, which is the primary intent of the DASH diet. Because the effects on BP were mixed in the articles included in this review, determining the best intervention duration for such interventions is important. Further investigation of the best practices related to the development and delivery of digital-based interventions to improve adherence to the DASH dietary pattern is warranted. Based on the findings of this review, digital-based and online delivery of DASH interventions may be successfully implemented across diverse settings, age groups, countries, and populations, but evidence-based approaches and digital engagement strategies must be considered in intervention design.

## Figures and Tables

**Table 1:** PICOS criteria for inclusion and exclusion of studies.

Parameter	Inclusion criteria	Exclusion criteria
Population	All age groups	N/A

Morbidities	With or without co-morbidities	N/A

Intervention type	All digitally delivered interventions based on dietary approaches to stop hypertension (DASH) or including a DASH component, which examine:	Non digitally formatted or delivered interventions
	- Measuring DASH scores	
	- Adherence to DASH	
	- Impact of DASH on nutrition behaviors	
	- Impact of DASH on nutrition-related health outcomes	

Outcomes	Blood pressure weight	Non-numeric/categorical assessment
	Blood lipid profile food intake	
	Diet quality	
	Food purchasing measures sodium intake	
	Overall quality of healthy eating index	
	Commitment in using and accessing the platform or the application	

Effectiveness of the DASH intervention	Change in scores for DASH adherence changes in blood pressure	N/A
	Changes in weight loss changes in sodium intake	
	Changes in quality of food purchasing and consumption	
	Changes in quality of healthy eating index	
	Changes in frequency of accessing and using the platform or the application	

Language	English	All other languages

Category study types	Peer-reviewed articles intervention-based studies	Nonpeer-reviewed articles commentaries
		Narratives communications conference papers white papers gray literature
		Similar article types protocols
		Feasibility studies without measured
		Outcomes reported

*Note:* N/A: not applicable.

**Table 2:** Summary of literature search (*N* = 24).

Authors, year/country	Quality score —/Ø/+	Target population/sample size	Type of study	Theory/framework	Intervention	Main results	Conclusion
Alnooh et al., 2024/Saudi Arabia	—	14 adults with HPTN	1 group, 8 week pretest and post-test	N/R	Use noon app daily for 8 weeks to enter all food/beverages consumed	Improvement in self-efficacy and DASH diet adherence not statistically significant; participants found noon app acceptable, suggestions included developing Arabic version, simplifying method of food log in	App was feasible, acceptable; studies needed to examine apps in promoting adherence to DASH diet, BP impact in Saudi Arabia
Darabi et al., 2024/Iran	Ø	88 adults aged 30–69 with HTN	Parallel group RCT × 12 weeks	Social cognitive theory	mHealth intervention IG: usual care + mHealth app with DASH and HTN education intervention; CG: usual care	Significant pre- and postdifferences in IG: SBP (*p*=0.0001), DBP (*p*=0.0001) compared to CG; 5 self-efficacy components improved significantly in IG (*p*=0.0001, *p*=0.0001, *p*=0.001, *p*=0.04, (*p*=0.000) compared to CG; no differences in DASH diet adherence (*p*=0.771)	Findings showed a mobile app for educating DASH diet, improving self-efficacy leads to better HPTN control, self-efficacy improvement
Eyles et al., 2017/New Zealand	Ø	66 adults with CVD; IG: 33; CG: 33	Two-arm, parallel, randomized controlled trial × 4 weeks	mHealth approach to behavior change	Use of salt switch smartphone app	Significant decreases in mean household salt purchases in IG compared to CG (*p*=0.03); no change in any secondary outcomes	SaltSwitch phone app effective in supporting people with CV disease to make lower salt food purchases; larger trial with longer follow-up needed
Glenn et al., 2019/Japan	Ø	559 Japanese adults ≥ 40 years	Single-arm, longitudinal pilot study × 16 weeks	Lifestyle and behavior change	Smartphone multidomain cognitive lifestyle intervention developed in U.S. for Japanese population	Significant relationships between engagement with app and nutrition score (*p*=0.03) and minutes exercised (*p*=0.01);	Program feasible as shown by commitment of participants, improvement in nutrition/PA; need larger evaluation with RCT
Hollis-Hansen et al., 2020/USA	—	60 mothers aged 28–53 with BMI > 24.9; *N* = 60, 4 groups, 15/group	2 × 2 factorial randomized pilot study × 1 week	NIH stage model of behavior change	Exploratory trial using the internet × 4 arms: -SET + DASH -EFT + SAFETY SET+	EFT group significantly increased in DASH diet score (*p* < 0.05) compared to SET group; no significant effect of DASH education nor DASH by EFT interactions	Dietary intake, food purchasing results must be replicated in larger samples
Jahan et al., 2020/Bangladesh	+	412 patients with HTN aged ≥ 35; IG: 204; CG: 208	Prospective RCT parallel-group trial × 5 months	Health behavior change	IG: SMS health information text messages + health education; CG: health education only	CG had significantly higher salt intake adherence rates (*p*=0.04), physical activity adherence rates (*p* < 0.03); salt intake in both groups improved significantly (*p* < 0.001); both groups decreased mean BP (*p* < 0.05), reported improved quality of life (*p* < 0.001)	Can improve intervention with more relevant/timely SMS text messages; community awareness encourages “low-salt culture”
Lesley, 2007/USA	Ø	78 African–American adults	Randomized two groups, multiple post-test design	Social problem-solving model	Two mobile education programs: IG: DASH app plus problem-solving training program; CG: DASH app alone	Intervention effect greatest for participants with BP screenings above normal	Problem-solving training combined with nutrition information may help African and Americans to deal more effectively with dietary problems
Miller et al., 2016/USA	+	123 African–American adults with HTN; IG: 62; CG: 61	Single-center RCT with two parallel arms × 8 weeks	N/R	IG: DASH plus coach-directed dietary advice via cell phone; CG: diet brochure plus debit account to purchase food	IG had significant increase in self-reported fruit/vegetable consumption, estimated intake of potassium, urine potassium excretion compared to CG (*p* < 0.05)	Larger trial needed to obtain a strong evidence about intervention benefit, BP effects
Miller et al., 2025/USA	+	301 adults with HPTN	RCT × 12 months	N/R	IG: digital health intervention (DHI) with commercially available app for daily dietary tracking data to compute DASH score, automated, tailored feedback; CG: tracked daily intake in app; access to DASH skills/materials	DASH scores not significantly different between arms at 6 months; IG had significantly greater 12-month changes in DASH score; between-group differences in 6 month changes insignificant for SBP, marginally significant for DBP	DHI had modest BP and DASH improvements; further research needed to understand utility of DHIs to promote DASH, identify interventions to support long-term behavior change
Moore et al., 2008/USA	Ø	2834 employees and spouses of EMC corporation	Longitudinal observational study × 12 months	N/R	DASH for health program with weekly internet articles about healthy nutrition	At 12 months, *n* = 735 (26%) still actively using program; subjects with BMI > 25 (*n* = 151) had significant weight change (*p* < 00.001); those with baseline HTN or pre-HTN had significant change in SBP (*p* < 0.001), but not DBP (*p*=0.16); visiting website more often led to greater BP/weight loss	Continuous use of internet delivered nutrition education program led to significant improvement in weight loss, BP diet; effective internet-delivered programs provide benefit to large numbers of subjects at low cost
Rich-Edwards et al., 2019/ USA	+	151 adult women with pre-eclampsia in past 5 years	RCT × 9 months	Social cognitive theory	IG: online educational modules, a community forum, communication with a lifestyle coach CG: Internet links to CVD risk reduction	IG had significantly greater knowledge of CVD risk factors (*p*=0.01), self-efficacy for healthy eating (*p*=0.03) compared to CG; no significant differences between groups for DASH adherence, or self-efficacy for/actual reported PA	Program improved CVD risk knowledge, self-efficacy to achieve a healthy diet; reduced physical inactivity among women with recent preeclampsia
Schiwal et al., 2020/USA	Ø	146 adults/general community; IG: 104; CG: 42	RCT × 6 months	Self-determination theory	Gray matters app targeted lifestyle risks For Alzheimer's disease, including DASH diet; IG: student health coach, activity tracker + app; CG: health coach + study website	IG motivation increased significantly (2.09 ± 4.82), compared to CG (*p*=0.003); vigorous PA increased in males with high IM compared to lower IM males (*p*=0.030). Diet Quality significantly improved in persons With high vs. low IM (*p*=0.038)	App improved IM, PA/diet quality for subgroups; future research should examine how IM moderates change across age groups, gender; other motivation associated with behavioral change
Serlachius et al., 2019/New Zealand	Ø	72 adults with gout; gout IG: 36; DASH IG: 36	RCT × 2 weeks	Behavioral self-management	2 week intervention with self-management or DASH app	Gout IG found app more engaging (*p*=0.003), informative (*p*=0.04) than DASH IG at 2 week follow-up; were no significant differences in self-care behaviors	Differences in engagement scores did not translate into Differences in self-care behaviors
Staffileno et al., 2018/USA	+	26 African–American women, age 18-45 with pre-HTN; DASH group: 14; PA group: 12	RCT × 12 weeks	Social cognitive theory, MI, behavioral self-management	Web-based, culturally relevant lifestyle intervention; IG: DASH online education; CG: PA, online education	Significant change in total DASH score (*p*=00.001) between groups; completion of activities 71%/48% in DASH/PA groups	eHealth platform feasible for improving PA, dietary behaviors in African–American women
Steen et al., 2022/USA	+	247 adults ≥ 1 CV risk factor; IG#1: 100; IG#2: 101; CG: 46	3 arm RCT with 3 months follow up	Individualized education sessions	Dietician delivered web and supermarket intervention; IG#1: point of purchase DASH nutrition education; IG#2: purchase DASH nutrition education, online shopping technologies/training; CG: Standard of care	DASH score significantly increased for the combined IG#1 and IG#2 compared to the CG (*p*=0.02).	Results suggest efficacy of supermarket-based, dietary interventions, modern online shopping tools in improving dietary quality; opportunity for academic investigators to collaborate with retailers to design and test comprehensive healthcare interventions
Steinberg et al., 2019/USA	+	306 adult patients with elevated cardiovascular risk	RCT × 12 months	Social cognitive theory	Digital weight loss intervention; IG: mobile app use, tailored diet feedback, weekly monitoring, PA CG: usual care	Improvements in IG DASH nutrient score (*p* < 0.001), weight loss (*p*=0.003) IG compared to CG; no difference in DASH food score between groups; no association found between DASH adherence/BP changes	Focusing on both calorie restriction and diet quality is recommended in interventions promoting weight loss
Steinberg et al., 2020/USA	+	59 adult women, BMI ≥ 18.5 and HTN CG: 29; IG: 30	RCT × 3 months	Health and behavior change model	DASH cloud intervention; IG: app diet tracking plus feedback on DASH adherence; CG: app diet tracking	No significant changes between IG and CG for mean days tracked (*p*=0.54), DASH score changes (*p*=0.75)	Digital health intervention feasible, produced high engagement
Sun et al., 2024/China	+	54 adults > 60 with primary HTN or currently taking HTN medication; IG: 23; CG: 31	2-arm RCT × 12 weeks	Behavior change wheel, integrating 19 behavior change frameworks	IG: received health behavioral digital intervention for hypertensive patients (HBDIHP) + regular care; CG: routine care	Significant changes in SBP (*p*=0.05), exercise time (*p*=0.03), medication adherence (*p*=0.02), BP monitoring frequency (*p*=0.046), learning performance (*p* < 0.001) between IG and CG	Program may have enhanced health outcomes, adherence to health behaviors; longer-term, larger-scale trial necessary to validate effectiveness
Svetkey et al., 2009/USA	+	32 physicians and 574 patients with HTN MD-I: 16; MC-C: 16 Pt-I: 185; Pt-C: 89	2 × 2 nested RCT × 18 months	N/R	MD-I: HTN training, lifestyle guidance, care; MD-C: usual care; Pt-I: behavior change education, lifestyle counseling; Pt-C: usual care + visit with provider for advice, written materials on lifestyle modifications	Largest effect was found in the combined MD-I/Pt-I group; significant decrease in SBP (*p*=0.0072) compared to all other groups	Combined physician/patient intervention lowers blood pressure; future research should focus on enhancing effectiveness/sustainability
Svetkey et al., 2015/USA	+	365 adults ages 18–35 with BMI ≥ 25; IG#1: (cell phone): IG#2: (cell phone plus coaching): CG: 123	RCT × 24 months	Social cognitive theory, transtheoretical model	mHealth intervention comparing DASH diet education delivered via cell phone to cell phone plus personal coaching	IG 2 participants lost significantly more weight than IG 1 and CG at 6 months (*p*=0.003), however, that result did not persist at months 12 or 24	Despite strong design, high engagement and retention, interventions did not lead to sustained weight loss; results sound cautionary note re mobile app delivery
Toro-Ramos et al., 2016/USA	Ø	50 adults with pre-HTN or HTN	Pre- and post-test pilot study × 24 weeks	MI, CBT	Mobile app intervention with human coaching	Significant reductions in weight (*p* < 0.001), DBP (*p*=0.004), HPN (*p* < 0.001); those completing ≥ 80% of program had significant decreases in SBP (*p* < 0.001), weight (*p* < 0.001)	Mobile delivery of intervention showed short-term potential to reduce HPN risk; need for longer-term studies
Van horn et al., 2018/USA	+	281 overweight or obese pregnant women; CG: 141; IG: 140	RCT × 24–36 weeks	MI	IG: DASH/PA phone app coaching sessions, reminders, newsletters, pedometer, text messages; CG: usual care	IG: DASH scores, HEI scores significantly improved (*p*=0.01, *p*=0.005, and *p*=0.002), lower gestational weight gain (*p*=0.01) compared to CG; no differences in birth weight, percentage body fat, or adverse pregnancy outcomes	Intervention led to better nutrient quality; improving DASH diet/PA adherence needed
Weerahandi et al., 2020/USA	—	17 adults 18–65, on HTN meds or stage 1 HTN	Single-arm pilot study × 4 months	MI	DASH mobile app intervention with health coach	No significant findings on primary parameters from pre- to postintervention.	High overall participant interaction with app suggests good tool for behavioral change
Youngiam and Therawiwat 2024/Thailand	+	80 adults 30–59 with pre-HPTN and high-sodium diet	2-Group RCT × 8 weeks	Integrated model of health literacy	IG: interactive app with 8 weekly activities	IG: NA consumption/DASH diet health literacy increased significantly (*p* < 0.001) and mean score of sodium consumption behavior, sodium intake, SBP, DBP systolic (*p* < 0.05); no change in CG	App is practical effective in reducing NA intake, BP; further testing in other populations/settings needed

*Note:* HTN: hypertension; N/R: not reported; SAFETY = food safety education.

Abbreviations: BMI, body mass index; BP, blood pressure; CBT, cognitive behavioral therapy; CG, control group; CVD, cardiovascular disease; DASH, Dietary Approaches to Stop Hypertension; DBP, diastolic blood pressure; EFT = episodic future thinking; HEI, Healthy Eating Index; IG, intervention group; IM, intrinsic motivation; MI, motivational interviewing; PA, physical activity; RCT, randomized controlled trial; SBP, systolic blood pressure; SET, standardized episodic thinking.

## Data Availability

The authors confirm that the data supporting the findings of this review are available within the article, figure, and tables.

## Supporting Information

Scopus Database

“online” [All Fields]; “app” [All Fields]; “digital” [All Fields]; computer” [All Fields]; “technology” [All Fields]; “internet” [All Fields]; “web-based” [All Fields]

AND

“Intervention” [All Fields]; “Program” [All Fields]; “education” [All Fields]

AND

“DASH diet” [All Fields]; “Dietary Approaches to Stop Hypertension dietary pattern” [All Fields]; “D.A.S.H diet” [All Fields]; AND

“hypertension” [All Fields]; “blood pressure” [All Fields]

## References

[1] Mills K. T., Stefanescu A., He J. (2020). The Global Epidemiology of Hypertension. *Nature Reviews Nephrology*.

[2] Mills K. T., Bundy J. D., Kelly T. N. (2016). Global Disparities of Hypertension Prevalence and Control. *Circulation*.

[3] World Health Organization (2023). *Hypertension*.

[4] Wu S., Xu Y., Zheng R. (2022). Hypertension Defined by 2017 ACC/AHA Guideline, Ideal Cardiovascular Health Metrics, and Risk of Cardiovascular Disease: A Nationwide Prospective Cohort Study. *The Lancet Regional Health-Western Pacific*.

[5] CDC (2024). About High Blood Pressure. https://www.cdc.gov/high-blood-pressure/about/index.html.

[6] Feigin V. L., Stark B. A., Johnson C. O. (2021). Global, Regional, and National Burden of Stroke and Its Risk Factors, 1990–2019: A Systematic Analysis for the Global Burden of Disease Study 2019. *The Lancet Neurology*.

[7] Muntner P., Hardy S. T., Fine L. J. (2020). Trends in Blood Pressure Control Among Us Adults With Hypertension, 1999–2000 to 2017–2018. *JAMA*.

[8] Rivier C. A., Renedo D. B., Sunmonu N. A., de Havenon A., Sheth K. N., Falcone G. J. (2024). Neighborhood Deprivation, Race, Ethnicity, and Undiagnosed Hypertension: Results From the all of US Research Program. *Hypertension*.

[9] Zhou B., Carrillo-Larco R. M., Danaei G. (2021). Worldwide Trends in Hypertension Prevalence and Progress in Treatment and Control From 1990 to 2019: A Pooled Analysis of 1201 Population-Representative Studies With 104 Million Participants. *The Lancet*.

[10] Mill J. G. (2019). Social Determinants of Hypertension. *Arquivos Brasileiros de Cardiologia*.

[11] Akinyelure O. P., Jaeger B. C., Oparil S. (2023). Social Determinants of Health and Uncontrolled Blood Pressure in a National Cohort of Black and White US Adults: The Regards Study. *Hypertension*.

[12] Leng B., Jin Y., Li G., Chen L., Jin N. (2015). Socioeconomic Status and Hypertension: A Meta-Analysis. *Journal of Hypertension*.

[13] Pescatello L. S., Buchner D. M., Jakicic J. M. (2019). Physical Activity to Prevent and Treat Hypertension: A Systematic Review. *Medicine & Science in Sports & Exercise*.

[14] Ndanuko R. N., Tapsell L. C., Charlton K. E., Neale E. P., Batterham M. J. (2016). Dietary Patterns and Blood Pressure in Adults: A Systematic Review and Meta-Analysis of Randomized Controlled Trials. *Advances in Nutrition*.

[15] Wang Z., Lu C., Wang Y. (2024). Association Between Ultra-Processed Foods Consumption and the Risk of Hypertension: An Umbrella Review of Systematic Reviews. *Hellenic Journal of Cardiology*.

[16] Wang M., Du X., Huang W., Xu Y. (2022). Ultra-Processed Foods Consumption Increases the Risk of Hypertension in Adults: A Systematic Review and Meta-Analysis. *American Journal of Hypertension*.

[17] Nardocci M., Polsky J. Y., Moubarac J. C. (2021). Consumption of Ultra-Processed Foods Is Associated With Obesity, Diabetes and Hypertension in Canadian Adults. *Canadian Journal of Public Health*.

[18] Theodoridis X., Chourdakis M., Chrysoula L. (2023). Adherence to the Dash Diet and Risk of Hypertension: A Systematic Review and Meta-Analysis. *Nutrients*.

[19] Filippou C. D., Tsioufis C. P., Thomopoulos C. G. (2020). Dietary Approaches to Stop Hypertension (Dash) Diet and Blood Pressure Reduction in Adults With and Without Hypertension: A Systematic Review and Meta-Analysis of Randomized Controlled Trials. *Advances in Nutrition*.

[20] Soltani S., Arablou T., Jayedi A., Salehi-Abargouei A. (2020). Adherence to the Dietary Approaches to Stop Hypertension (Dash) Diet in Relation to All-Cause and Cause-Specific Mortality: A Systematic Review and Dose-Response Meta-Analysis of Prospective Cohort Studies. *Nutrition Journal*.

[21] Murtaugh M. A., Beasley J. M., Appel L. J. (2018). Relationship of Sodium Intake and Blood Pressure Varies With Energy Intake: Secondary Analysis of the Dash (Dietary Approaches to Stop hypertension)-Sodium Trial. *Hypertension*.

[22] Hunter R. W., Dhaun N., Bailey M. A. (2022). The Impact of Excessive Salt Intake on Human Health. *Nature Reviews Nephrology*.

[23] Soltani S., Shirani F., Chitsazi M. J., -Abargouei A. (2016). The Effect of Dietary Approaches to Stop Hypertension (Dash) Diet on Weight and Body Composition in Adults: A Systematic Review and Meta-Analysis of Randomized Controlled Clinical Trials. *Obesity Reviews*.

[24] Benajiba N., Dodge E., Khaled M. B., Chavarria E. A., Sammartino C. J., Aboul-Enein B. H. (2022). Technology-Based Nutrition Interventions Using the Mediterranean Diet: A Systematic Review. *Nutrition Reviews*.

[25] Villinger K., Wahl D. R., Boeing H., Schupp H. T., Renner B. (2019). The Effectiveness of App-Based Mobile Interventions on Nutrition Behaviours and Nutrition-Related Health Outcomes: A Systematic Review and Meta-Analysis. *Obesity Reviews*.

[26] Zarnowiecki D., Mauch C. E., Middleton G. (2020). A Systematic Evaluation of Digital Nutrition Promotion Websites and Apps for Supporting Parents to Influence Children’s Nutrition. *International Journal of Behavioral Nutrition and Physical Activity*.

[27] Rose T., Barker M., Maria Jacob C. (2017). A Systematic Review of Digital Interventions for Improving the Diet and Physical Activity Behaviors of Adolescents. *Journal of Adolescent Health*.

[28] Beleigoli A. M., Andrade A. Q., Cançado A. G., Paulo M. N., Diniz M. D. F. H., Ribeiro A. L. (2019). Web-Based Digital Health Interventions for Weight Loss and Lifestyle Habit Changes in Overweight and Obese Adults: Systematic Review and Meta-Analysis. *Journal of Medical Internet Research*.

[29] Oh H., Rizo C., Enkin M., Jadad A., Powell J., Pagliari C. (2005). What is Ehealth (3): A Systematic Review of Published Definitions. *Journal of Medical Internet Research*.

[30] Hallberg D., Salimi N. (2020). Qualitative and Quantitative Analysis of Definitions of e-Health and m-Health. *Healthcare Informatics Research*.

[31] Burrows T. L., Khambalia A. Z., Perry R. (2015). Great App-Eal But Not There Yet: A Review of Iphone Nutrition Applications Relevant to Child Weight Management. *Nutrition and Dietetics*.

[32] Enam A., Torres-Bonilla J., Eriksson H. (2018). Evidence-Based Evaluation of Ehealth Interventions: Systematic Literature Review. *Journal of Medical Internet Research*.

[33] Vandelanotte C., Müller A. M., Short C. E. (2016). Past, Present, and Future of Ehealth and Mhealth Research to Improve Physical Activity and Dietary Behaviors. *Journal of Nutrition Education and Behavior*.

[34] Oakley-Girvan I., Yunis R., Longmire M., Ouillon J. S. (2022). What Works Best to Engage Participants in Mobile App Interventions and e-Health: A Scoping Review. *Telemedicine Journal and e-Health: The Official Journal of the American Telemedicine Association*.

[35] McGarrigle L., Todd C. (2020). Promotion of Physical Activity in Older People Using Mhealth and Ehealth Technologies: Rapid Review of Reviews. *Journal of Medical Internet Research*.

[36] Singh B., Ahmed M., Staiano A. E. (2024). A Systematic Umbrella Review and Meta-Meta-analysis of Ehealth and Mhealth Interventions for Improving Lifestyle Behaviours. *NPJ Digital Medicine*.

[37] Fiedler J., Eckert T., Wunsch K., Woll A. (2020). Key Facets to Build up Ehealth and Mhealth Interventions to Enhance Physical Activity, Sedentary Behavior and Nutrition in Healthy Subjects—An Umbrella Review. *BMC Public Health*.

[38] Tallon J. M., Saavedra Dias R., Costa A. M. (2021). Impact of Technology and School-Based Nutrition Education Programs on Nutrition Knowledge and Behavior During Adolescence—A Systematic Review. *Scandinavian Journal of Educational Research*.

[39] Bergevi J., Andermo S., Woldamanuel Y., Johansson U. B., Hagströmer M., Rossen J. (2022). User Perceptions of Ehealth and Mhealth Services Promoting Physical Activity and Healthy Diets: Systematic Review. *JMIR Human Factors*.

[40] Bruce C., Harrison P., Giammattei C. (2020). Evaluating Patient-Centered Mobile Health Technologies: Definitions, Methodologies, and Outcomes. *JMIR mHealth and uHealth*.

[41] Robert C., Erdt M., Lee J., Cao Y., Naharudin N. B., Theng Y. L. (2021). Effectiveness of Ehealth Nutritional Interventions for Middle-Aged and Older Adults: Systematic Review and Meta-Analysis. *Journal of Medical Internet Research*.

[42] Duan Y., Shang B., Liang W., Du G., Yang M., Rhodes R. E. (2021). Effects of Ehealth-Based Multiple Health Behavior Change Interventions on Physical Activity, Healthy Diet, and Weight in People With Noncommunicable Diseases: Systematic Review and Meta-Analysis. *Journal of Medical Internet Research*.

[43] Armaou M., Araviaki E., Musikanski L. (2020). Ehealth and Mhealth Interventions for Ethnic Minority and Historically Underserved Populations in Developed Countries: An Umbrella Review. *International Journal of Community Well-Being*.

[44] Lee E. W., McCloud R. F., Viswanath K. (2022). Designing Effective Ehealth Interventions for Underserved Groups: Five Lessons From a Decade of Ehealth Intervention Design and Deployment. *Journal of Medical Internet Research*.

[45] Appel L. J., Moore T. J., Obarzanek E. (1997). A Clinical Trial of the Effects of Dietary Patterns on Blood Pressure. *New England Journal of Medicine*.

[46] Tricco A. C., Lillie E., Zarin W. (2018). Prisma Extension for Scoping Reviews (prisma-scr): Checklist and Explanation. *Annals of Internal Medicine*.

[47] Arksey H., O’Malley L. (2005). Scoping Studies: Towards a Methodological Framework. *International Journal of Social Research Methodology*.

[48] https://www.andeal.org/vault/2440/web/files/2016_April_EA_Manual.pdf.

[49] Van Horn L., Peaceman A., Kwasny M. (2018). Dietary Approaches to Stop Hypertension Diet and Activity to Limit Gestational Weight: Maternal Offspring Metabolics Family Intervention Trial, A Technology Enhanced Randomized Trial. *American Journal of Preventive Medicine*.

[50] Svetkey L. P., Batch B. C., Lin P. H. (2015). Cell Phone Intervention for You (City): A Randomized, Controlled Trial of Behavioral Weight Loss Intervention for Young Adults Using Mobile Technology. *Obesity*.

[51] Svetkey L. P., Pollak K. I., Yancy W. S. (2009). Hypertension Improvement Project: Randomized Trial of Quality Improvement for Physicians and Lifestyle Modification for Patients. *Hypertension*.

[52] Sun T., Xu X., Ding Z. (2024). Development of a Health Behavioral Digital Intervention for Patients With Hypertension Based on an Intelligent Health Promotion System and Wechat: Randomized Controlled Trial. *JMIR mHealth and uHealth*.

[53] Steinberg D. M., Kay M. C., Svetkey L. P. (2020). Feasibility of a Digital Health Intervention to Improve Diet Quality Among Women With High Blood Pressure: Randomized Controlled Feasibility Trial. *JMIR mHealth and uHealth*.

[54] Steinberg D., Kay M., Burroughs J., Svetkey L. P., Bennett G. G. (2019). The Effect of a Digital Behavioral Weight Loss Intervention on Adherence to the Dietary Approaches to Stop Hypertension (Dash) Dietary Pattern in Medically Vulnerable Primary Care Patients: Results From a Randomized Controlled Trial. *Journal of the Academy of Nutrition and Dietetics*.

[55] Steen D. L., Helsley R. N., Bhatt D. L. (2022). Efficacy of Supermarket and Web-Based Interventions for Improving Dietary Quality: A Randomized, Controlled Trial. *Nature Medicine*.

[56] Staffileno B. A., Tangney C. C., Fogg L. (2018). Favorable Outcomes Using an Ehealth Approach to Promote Physical Activity and Nutrition Among Young African American Women. *Journal of Cardiovascular Nursing*.

[57] Serlachius A., Schache K., Kieser A., Arroll B., Petrie K., Dalbeth N. (2019). Association Between User Engagement of a Mobile Health App for Gout and Improvements in Self-Care Behaviors: Randomized Controlled Trial. *JMIR mHealth and uHealth*.

[58] Schiwal A. T., Fauth E. B., Wengreen H., Norton M. (2020). The Gray Matters App Targeting Health Behaviors Associated With Alzheimer’s Risk: Improvements in Intrinsic Motivation and Impact on Diet Quality and Physical Activity. *The Journal of Nutrition, Health & Aging*.

[59] Rich-Edwards J. W., Stuart J. J., Skurnik G. (2019). Randomized Trial to Reduce Cardiovascular Risk in Women With Recent Preeclampsia. *Journal of Women’s Health*.

[60] Lesley M. L. (2007). Social Problem Solving Training for African Americans: Effects on Dietary Problem Solving Skill and Dash Diet-Related Behavior Change. *Patient Education and Counseling*.

[61] Jahan Y., Rahman M. M., Faruque A. S. G. (2020). Awareness Development and Usage of Mobile Health Technology Among Individuals With Hypertension in a Rural Community of Bangladesh: Randomized Controlled Trial. *Journal of Medical Internet Research*.

[62] Hollis-Hansen K., Seidman J., O’Donnell S., Epstein L. H. (2020). Mothers’ Dash Diet Adherence and Food Purchases After Week-Long Episodic Future Thinking Intervention. *Appetite*.

[63] Eyles H., McLean R., Neal B. (2017). A Salt-Reduction Smartphone App Supports Lower-Salt Food Purchases for People With Cardiovascular Disease: Findings From the Saltswitch Randomised Controlled Trial. *European Journal of Preventive Cardiology*.

[64] Darabi Z., Araban M., Azizi A., Ahmadi Angali K., Borazjani F. (2023). The Effectiveness of a Mobile Phone Education Method Based on Self-Efficacy and Dash Diet Among Patients With High Blood Pressure: A Randomized Controlled Trial. *Jundishapur Journal of Chronic Disease Care*.

[65] Miller E. R., Cooper L. A., Carson K. A. (2016). A Dietary Intervention in Urban African Americans: Results of the “Five plus Nuts and Beans” Randomized Trial. *American Journal of Preventive Medicine*.

[66] Glenn J., Madero E. N., Gray M. (2019). Engagement With a Digital Platform for Multimodal Cognitive Assessment and Multidomain Intervention in a Japanese Population: Pilot, Quasi-Experimental, Longitudinal Study. *JMIR mHealth and uHealth*.

[67] Moore T. J., Alsabeeh N., Apovian C. M. (2008). Weight, Blood Pressure, and Dietary Benefits After 12 Months of a Web-Based Nutrition Education Program (Dash for Health): Longitudinal Observational Study. *Journal of Medical Internet Research*.

[68] Toro-Ramos T., Kim Y., Wood M. (2017). Efficacy of a Mobile Hypertension Prevention Delivery Platform With Human Coaching. *Journal of Human Hypertension*.

[69] Weerahandi H., Paul S., Quintiliani L. M., Chokshi S., Mann D. M. (2020). A Mobile Health Coaching Intervention for Controlling Hypertension: Single-Arm Pilot Pre-Post Study. *JMIR Formative Research*.

[70] Alnooh G., AlTamimi J. Z., Williams E. A., Hawley M. S. (2024). An Investigation of the Feasibility and Acceptability of Using a Commercial Dash (Dietary Approaches to Stop Hypertension) App in People With High Blood Pressure: Mixed Methods Study. *JMIR Formative Research*.

[71] Youngiam W., Therawiwat M. (2023). Enhancing Health Literacy Through “I Watch Sodium” Application Among Prehypertension University Staff: A Quasi-Experimental Study. *Pacific Rim International Journal of Nursing Research*.

[72] Miller H. N., Askew S., Berger M. B. (2025). Effects of a Digital Intervention to Improve Dash and Blood Pressure Among us Adults. *Hypertension*.

[73] Zou P., Stinson J., Parry M., Dennis C. L., Yang Y., Lu Z. (2020). A Smartphone App (Mdashna-cc) to Support Healthy Diet and Hypertension Control for Chinese Canadian Seniors: Protocol for Design, Usability and Feasibility Testing. *JMIR Research Protocols*.

[74] Miller H. N., Berger M. B., Askew S. (2021). The Nourish Protocol: A Digital Health Randomized Controlled Trial to Promote the Dash Eating Pattern Among Adults With Hypertension. *Contemporary Clinical Trials*.

[75] Gardiner P., McGonigal L., Villa A. (2022). Our Whole Lives for Hypertension and Cardiac Risk Factors-Combining a Teaching Kitchen Group Visit With a Web-Based Platform: Feasibility Trial. *JMIR Formative Research*.

[76] Jerome G. J., Lisman P. J., Dalcin A. T., Clark A. (2020). Weight Management Program for First Responders: Feasibility Study and Lessons Learned. *Work*.

[77] Sacks N., Cabral H., Kazis L. E. (2009). A Web-Based Nutrition Program Reduces Health Care Costs in Employees With Cardiac Risk Factors: Before and After Cost Analysis. *Journal of Medical Internet Research*.

[78] Sheeran P., Klein W. M., Rothman A. J. (2017). Health Behavior Change: Moving From Observation to Intervention. *Annual Review of Psychology*.

[79] Conner M., Norman P. (2017). Health Behaviour: Current Issues and Challenges. *Psychology and Health*.

[80] Michie S., West R., Sheals K., Godinho C. A. (2018). Evaluating the Effectiveness of Behavior Change Techniques in Health-Related Behavior: A Scoping Review of Methods Used. *Translational Behavioral Medicine*.

[81] Rubinelli S., Diviani N. (2020). The Bases of Targeting Behavior in Health Promotion and Disease Prevention. *Patient Education and Counseling*.

[82] Carey R. N., Connell L. E., Johnston M. (2019). Behavior Change Techniques and Their Mechanisms of Action: A Synthesis of Links Described in Published Intervention Literature. *Annals of Behavioral Medicine: A Publication of the Society of Behavioral Medicine*.

[83] Michie S., Yardley L., West R., Patrick K., Greaves F. (2017). Developing and Evaluating Digital Interventions to Promote Behavior Change in Health and Health Care: Recommendations Resulting From an International Workshop. *Journal of Medical Internet Research*.

[84] Ashton L. M., Sharkey T., Whatnall M. C. (2019). Effectiveness of Interventions and Behaviour Change Techniques for Improving Dietary Intake in Young Adults: A Systematic Review and Meta-Analysis of Rcts. *Nutrients*.

[85] Murimi M. W., Kanyi M., Mupfudze T., Amin M. R., Mbogori T., Aldubayan K. (2017). Factors Influencing Efficacy of Nutrition Education Interventions: A Systematic Review. *Journal of Nutrition Education and Behavior*.

[86] Mummah S. A., Robinson T. N., King A. C., Gardner C. D., Sutton S. (2016). Ideas (Integrate, Design, Assess, and Share): A Framework and Toolkit of Strategies for the Development of More Effective Digital Interventions to Change Health Behavior. *Journal of Medical Internet Research*.

[87] Bully P., Sánchez Á., Zabaleta-del-Olmo E., Pombo H., Grandes G. (2015). Evidence From Interventions Based on Theoretical Models for Lifestyle Modification (Physical Activity, Diet, Alcohol and Tobacco Use) in Primary Care Settings: A Systematic Review. *Preventive Medicine*.

[88] Morgan P. J., Young M. D., Smith J. J., Lubans D. R. (2016). Targeted Health Behavior Interventions Promoting Physical Activity: A Conceptual Model. *Exercise and Sport Sciences Reviews*.

[89] Wilson K., Senay I., Durantini M. (2015). When It Comes to Lifestyle Recommendations, More is Sometimes Less: A Meta-Analysis of Theoretical Assumptions Underlying the Effectiveness of Interventions Promoting Multiple Behavior Domain Change. *Psychological Bulletin*.

[90] Samdal G. B., Eide G. E., Barth T., Williams G., Meland E. (2017). Effective Behaviour Change Techniques for Physical Activity and Healthy Eating in Overweight and Obese Adults; Systematic Review and Meta-Regression Analyses. *International Journal of Behavioral Nutrition and Physical Activity*.

[91] Rathbone A. L., Prescott J. (2017). The Use of Mobile Apps and Sms Messaging as Physical and Mental Health Interventions: Systematic Review. *Journal of Medical Internet Research*.

[92] Direito A., Carraça E., Rawstorn J., Whittaker R., Maddison R. (2017). Mhealth Technologies to Influence Physical Activity and Sedentary Behaviors: Behavior Change Techniques, Systematic Review and Meta-Analysis of Randomized Controlled Trials. *Annals of Behavioral Medicine*.

[93] Short C. E., DeSmet A., Woods C. (2018). Measuring Engagement in Ehealth and Mhealth Behavior Change Interventions: Viewpoint of Methodologies. *Journal of Medical Internet Research*.

[94] Milne-Ives M., Lam C., De Cock C., Van Velthoven M. H., Meinert E. (2020). Mobile Apps for Health Behavior Change in Physical Activity, Diet, Drug and Alcohol Use, and Mental Health: Systematic Review. *JMIR mHealth and uHealth*.

[95] O’Connor H., Willcox J. C., de Jersey S., Wright C., Wilkinson S. A. (2024). Digital Preconception Interventions Targeting Weight, Diet and Physical Activity: A Systematic Review. *Nutrition and Dietetics*.

[96] Roberts A. L., Fisher A., Smith L., Heinrich M., Potts H. W. W. (2017). Digital Health Behaviour Change Interventions Targeting Physical Activity and Diet in Cancer Survivors: A Systematic Review and Meta-Analysis. *Journal of Cancer Survivorship*.

[97] Chatterjee A., Prinz A., Gerdes M., Martinez S. (2021). Digital Interventions on Healthy Lifestyle Management: Systematic Review. *Journal of Medical Internet Research*.

[98] Coughlin S. S., Whitehead M., Sheats J. Q., Mastromonico J., Hardy D., Smith S. A. (2015). Smartphone Applications for Promoting Healthy Diet and Nutrition: A Literature Review. *Jacobs Journal of Food and Nutrition*.

[99] Ryan K., Dockray S., Linehan C. (2019). A Systematic Review of Tailored Ehealth Interventions for Weight Loss. *Digit Health*.

[100] Van Rhoon L., Byrne M., Morrissey E., Murphy J., McSharry J. (2020). A Systematic Review of the Behaviour Change Techniques and Digital Features in Technology-Driven Type 2 Diabetes Prevention Interventions. *Digit Health*.

[101] Yardley L., Spring B. J., Riper H. (2016). Understanding and Promoting Effective Engagement With Digital Behavior Change Interventions. *American Journal of Preventive Medicine*.

[102] Yardley L., Morrison L., Bradbury K., Muller I. (2015). The Person-Based Approach to Intervention Development: Application to Digital Health-Related Behavior Change Interventions. *Journal of Medical Internet Research*.

[103] Klonoff D. C. (2019). Behavioral Theory: The Missing Ingredient for Digital Health Tools to Change Behavior and Increase Adherence. *Journal of Diabetes Science and Technology*.

[104] Borghouts J., Eikey E., Mark G. (2021). Barriers to and Facilitators of User Engagement With Digital Mental Health Interventions: Systematic Review. *Journal of Medical Internet Research*.

