# Supplementary material for: Digital-Based Nutrition Interventions Employing the Dietary Approaches to Stop Hypertension (DASH) Diet: A Systematic Scoping Review
Source: Int J Hypertens. 2025 Aug 30;2025:6175223. doi: 10.1155/ijhy/6175223 (PMC12413951; doi:10.1155/ijhy/6175223)
Supplement: Supporting Information — Additional supporting information can be found online in the Supporting Information section. [file 6175223.f1.docx]

**Supplementary material**

***Scopus Database***

“online” [All Fields]; “app” [All Fields]; “digital” [All Fields]; computer” [All Fields]; “technology” [All Fields]; “internet” [All Fields]; “web-based” [All Fields]

AND

“Intervention” [All Fields]; “Program” [All Fields]; “education” [All Fields]

AND

“DASH diet” [All Fields]; “Dietary Approaches to Stop Hypertension dietary pattern” [All Fields]; “D.A.S.H diet” [All Fields];

AND

“hypertension” [All Fields]; “blood pressure” [All Fields]
